# Supplementary material for: The genome sequence of the Lesser Horseshoe Bat, Rhinolophus hipposideros (Bechstein, 1800) (Chiroptera: Rhinolophidae)
Source: Wellcome Open Res. 2026 Apr 10;11:212. [Version 1] doi: 10.12688/wellcomeopenres.26210.1 (PMC13090764; doi:10.12688/wellcomeopenres.26210.1)
Supplement: Peer review history

# Reviewer report: Reviewer response for version 1

**DOI:** 10.21956/wellcomeopenres.28868.r153216

**Published:** 2026-04-23

**Authors:** Michael Hiller

**Affiliations:** 1 Senckenberg Research Institute, Frankfurt, Germany

This data notes represents a high-quality haplotype resolved genome assembly of *Rhinolophus hipposideros.*

 The data note comprises a detailed introduction with a lot of useful information, and an accurate description of all steps to generate the genomic and transcriptomic data and to assemble the genome. 

 I only have one major question. 

 Is it not clear to me why GCA_964194185.1 is presented as the main haplotype, which has a low contig N50 of 2,014,157 bp, potentially because of an adapter trimming issue in the pipeline. 

 The original hap2.1 (GCA_964194215.1) also had a contig N50 of 1,922,115 bp, but this was later improved in the hap2.2 version (GCA_964194215.2) to 44 Mb. 

 Since hap2.2 was likely generated by a new haplotype-resolved assembly and dual curation, I wonder if the corresponding hap1.2 will also be released. Otherwise, the assembly methods need to be adjusted, as these 2 haplotypes most likely come from different assembly attempts.

 Also, because the TOGA2-inferred gene completeness of hap2.2 (GCA_964194215.2) is noticeably higher than hap1.1 (GCA_964194185.1), it would make sense to present hap2.2 as more complete primary hap1 for this species, even if hap1.1 received an Ensembl gene annotation. 

Are sufficient details of methods and materials provided to allow replication by others?

Yes

Is the rationale for creating the dataset(s) clearly described?

Yes

Are the datasets clearly presented in a useable and accessible format?

Yes

Are the protocols appropriate and is the work technically sound?

Yes

Reviewer Expertise:

comparative genomics, genome assembly

I confirm that I have read this submission and believe that I have an appropriate level of expertise to state that I do not consider it to be of an acceptable scientific standard, for reasons outlined above.

# Reviewer report: Reviewer response for version 1

**DOI:** 10.21956/wellcomeopenres.28868.r153220

**Published:** 2026-04-20

**Authors:** Jiang Feng

**Affiliations:** 1 Northeast Normal University, Changchun, China

This manuscript presents a high-quality, chromosome-level genome assembly of Rhinolophus hipposideros, a species characterized by chromosomal polymorphism and ecological sensitivity but lacking a well-established reference genome. The work uses a combined strategy of PacBio HiFi and Hi-C and RNA-seq, with complete procedures for haplotype-resolved assembly, mitochondrial genome assembly, and quality evaluation, which meets current standards for vertebrate reference genomes. Overall, the manuscript is scientifically sound and requires only minor revisions to improve clarity, consistency, and completeness.

 1.The abstract does not clearly state that this genome represents the first chromosome-level, haplotype-resolved genome for this species, so it is recommended to add this statement to emphasize its uniqueness.

 2.There is a conflict between “the sample is male” in the last of the Background and “adult female” in the Methods and Abstract. This inconsistency must be corrected throughout the manuscript.

 3. Please add the local collection permit number and ethical approval number in Cyprus, listing only the responsible government departments is insufficient.

 4. Please specify the homologous species set and pipeline version used for genome annotation, and the prediction criteria for non-coding genes.

 5. The sampling location is described inconsistently as “Cyprus, University of Cyprus”(Methods) and “Kalopanayiotis, Cyprus”(Background). These should be unified.

 6. The binomial name Rhinolophus hipposideros is not italicized in the Keywords. After its first mention, the abbreviation R. hipposideros should be used consistently in italics.

 7. The Methods mention that multiple tissues were sampled. Please explicitly list all tissues collected and used in this study.

Are sufficient details of methods and materials provided to allow replication by others?

Yes

Is the rationale for creating the dataset(s) clearly described?

Yes

Are the datasets clearly presented in a useable and accessible format?

Yes

Are the protocols appropriate and is the work technically sound?

Yes

Reviewer Expertise:

Animal Behavioral Ecology, Animal Molecular Ecology

I confirm that I have read this submission and believe that I have an appropriate level of expertise to confirm that it is of an acceptable scientific standard.

# Reviewer report: Reviewer response for version 1

**DOI:** 10.21956/wellcomeopenres.28868.r153218

**Published:** 2026-04-17

**Authors:** Xiuguang Mao

**Affiliations:** 1 East China Normal University, Shanghai, China

This manuscript presents a high-quality chromosome-scale genome assembly for *R. hipposideros*, which provides valuable insights into chromosomal evolution within this species complex, characterized by diploid numbers of 2n = 54, 56, and 58. The assembly and the associated comparative analyses represent a meaningful contribution to our understanding of karyotypic diversification in this group.

 For the current version of the manuscript, I have one minor comment:

 In the main text, the authors state that the BUSCO assessment was performed using the metazoa_odb10 reference set (n = 954). However, in Figure 5, the BUSCO database is reported as laurasiatheria_odb10 (n = 12,234). Please verify which dataset was actually used in the analysis and update both the text and the figure accordingly.

Are sufficient details of methods and materials provided to allow replication by others?

Yes

Is the rationale for creating the dataset(s) clearly described?

Yes

Are the datasets clearly presented in a useable and accessible format?

Yes

Are the protocols appropriate and is the work technically sound?

Yes

Reviewer Expertise:

Genome evolution, bats, phylogeny

I confirm that I have read this submission and believe that I have an appropriate level of expertise to confirm that it is of an acceptable scientific standard.

# Reviewer report: Reviewer response for version 1

**DOI:** 10.21956/wellcomeopenres.28868.r153214

**Published:** 2026-04-16

**Authors:** Graham Etherington

**Affiliations:** 1 Earlham Institute, Norwich, UK

The authors present the first annotated genome assembly for the lesser horseshoe bat. The manuscript clearly describes both the methods employed and the results obtained from the assembly, and provides a thorough and engaging overview of the species’ evolutionary and ecological background. Overall, the study is well presented. I have, however, a few minor suggestions that may further improve the clarity and completeness of the manuscript:

1. The term *“western Palaearctic”* should be capitalised as *“Western Palaearctic”*.
2. It would be helpful to include the common name ( *Mehely’s horseshoe bat*) when first mentioning *Rhinolophus mehelyi*.
3. The authors may wish to clarify whether the 29 chromosomal pseudomolecules correspond to the expected karyotype of the sequenced individual.
4. The manuscript refers to the “distinctive rDNA organisation patterns in *Rhinolophus hipposideros*”, but this point is not expanded upon. A brief explanation of what makes this organisation distinctive would improve reader understanding.

Are sufficient details of methods and materials provided to allow replication by others?

Yes

Is the rationale for creating the dataset(s) clearly described?

Yes

Are the datasets clearly presented in a useable and accessible format?

Yes

Are the protocols appropriate and is the work technically sound?

Yes

Reviewer Expertise:

Conservation genomics, genome assembly, vertebrate genomics.

I confirm that I have read this submission and believe that I have an appropriate level of expertise to confirm that it is of an acceptable scientific standard.
